# Vascular variants in seed plants—a developmental perspective

**DOI:** 10.1093/aobpla/plad036

**Published:** 2023-07-12

**Authors:** Israel L Cunha Neto

**Affiliations:** School of Integrative Plant Sciences and L.H. Bailey Hortorium, Cornell University, 237 Mann Dr, Ithaca, NY 14853, USA

**Keywords:** cambium, development, ectopic cambia, evolution, meristems, plant anatomy, procambium, vascular tissue

## Abstract

Over centuries of plant morphological research, biologists have enthusiastically explored how distinct vascular arrangements have diversified. These investigations have focused on the evolution of steles and secondary growth and examined the diversity of vascular tissues (xylem and phloem), including atypical developmental pathways generated through modifications to the typical development of ancestral ontogenies. A shared vernacular has evolved for communicating on the diversity of alternative ontogenies in seed plants. Botanists have traditionally used the term ‘anomalous secondary growth’ which was later renamed to ‘cambial variants’ by late Dr. Sherwin Carlquist (1988). However, the term ‘cambial variants’ can be vague in meaning since it is applied for developmental pathways that do not necessarily originate from cambial activity. Here, we review the ‘cambial variants’ concept and propose the term ‘vascular variants’ as a more inclusive overarching framework to interpret alternative vascular ontogenies in plants. In this framework, vascular variants are defined by their developmental origin (instead of anatomical patterns), allowing the classification of alternative vascular ontogenies into three categories: (i) *procambial variants*, (ii) *cambial variants* and (iii) *ectopic cambia*. Each category includes several anatomical patterns. Vascular variants, which represent broader developmental based groups, can be applied to both extant and fossil plants, and thereby offer a more adequate term from an evolutionary perspective. An overview of the developmental diversity and phylogenetic distribution of vascular variants across selected seed plants is provided. Finally, this viewpoint discusses the evolutionary implications of vascular variants.

## Teaser

‘Vascular variant’ is the developmental process by which atypical vascular growth emerges in a lineage in relation to their putative ancestor. These atypical ontogenies emerge from atypical procambium, cambium and *de novo* cambium patterning, outlining three developmental based categories: procambial variants, cambial variants and ectopic cambia. In turn, each category of vascular variants includes several anatomical patterns that are predictably conserved within a given taxon and, therefore, useful in the species description. These atypical—and frequently complex ontogenies—develop in stems and roots of all major lineages of seed plants, outlining a striking case of convergent evolution. The vascular variants' framework illuminates the evolution of the development of the massive diversity of vascular ontogenies in distantly related plants and promises to facilitate comparative studies that may be informative at various biological scales with meaningful evolutionary implications.

## Background

There are more than 300 000 living species of tracheophytes (vascular plants) and the occurrence of secondary growth through a single bifacial vascular cambium is evidenced in thousands of species, particularly in seed plants (Spicer and Groover 2010). However, modifications of this ‘typical’ vascular development arise in many ways (Haberlandt 1914; Esau 1965), generating a myriad of ‘alternative patterns of vascular growth’ (Carlquist 1991). These alternative anatomies are seen particularly via the presentation of distinct stem ontogenies in comparison to the putative ancestral development of seed plants. The ancestor of seed plants likely had a stem ontogeny with a combination of a ‘typical’ eustele (although distinct stelar configurations are also recognized in early seed plants: Wang and Liu 2015), and homogeneous amounts of wood (secondary xylem) and inner bark (secondary phloem) produced by a single bifacial cambium (Simpson 2019; Onyenedum and Pace 2021). Although most extant gymnosperms and angiosperms display that conserved trajectory (= *typical growth*), some lineages have lost the eustele and/or the bifacial cambium, and others have evolved ‘alternative patterns of vascular growth’. These vascular modifications have been reported in multiple lineages of fossil and extant lignophytes, predominantly among angiosperms (Carlquist 1991; Bodnar and Coturel 2012; Decombeix *et al.* 2014; Angyalossy *et al.* 2015). In these alternative anatomies, the timing, spatial distribution, and relative abundance of xylem and phloem formed by vascular meristems are drastically altered, generating stems and roots with unusual shapes and arrangements of vascular tissues.

For many years, ‘anomalous secondary growth’ (Esau 1965) has been the most prevalent term in English to describe alternative vascular ontogenies in plants, though many other terms have existed **[see**  **Supporting Information—Table S1****]**. In 1988, Dr. Sherwin Carlquist coined the term ‘cambial variants’ while pointing out the misleading impression of a disorderly action in the antecedent term ‘anomalous secondary growth’ (Carlquist 1991, 2001). Surprisingly, Carlquist’s definition—and those that post-dated him (e.g. Spicer and Groover 2010; Angyalossy *et al.* 2012, 2015)—focusses on cambial activity and modifications during secondary growth, with no reference to procambial patterning (primary growth), despite the multiple reports on modifications in procambial organization in both fossil and extant plant lineages (Worsdell 1906; Esau 1965; Beck 2010). Because alternative vascular ontogenies may originate from modifications in procambium patterning, the term ‘cambial variant’ is not the most appropriate to broadly describe these phenomena, confusing our scientific understanding of this concept.

An important evolutionary perspective can be gained by devising morphological concepts that reflect how organisms evolve through changes in ontogeny. In an effort to better define ontogenies originating in an unusual procambial organization, as opposed to the cambial origin, the developmental stem anatomy of species across various families of extant angiosperms will be explored. The term ‘vascular variants’ is proposed to supplant ‘cambial variants’, as it more broadly describes alternative ontogenies within the context of development and evolution.

## What Are Vascular Variants?

**Figure 1.**
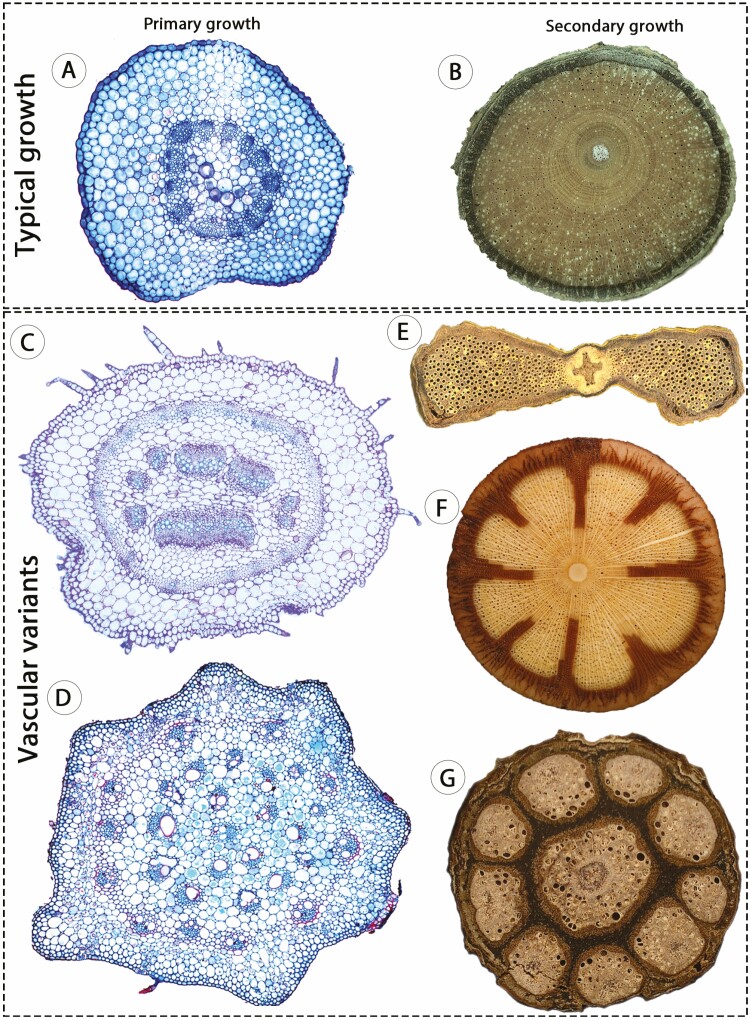
Stem vascular arrangements in angiosperms illustrating the typical growth and vascular variants. (A) Typical eustele; *Portulaca halimoides,* Portulacaceae; courtesy of Thaíla V. A. Santos. (B) Regular secondary growth; *Paullinia coriacea,* Sapindaceae; courtesy of Robson G. Silva. (C) Eustele with medullary bundles (in the centre of the pith) and continuous procambium forming additional vascular bundles in the periphery; *Allionia incarnata,* Nyctaginaceae. (D) Atactostele; *Commelina platyphylla,* Commelinaceae; courtesy of Ricardo S. B. Vita. (E) Non-cylindrical stem resulting from atypical cambial activity (single cambium); Schnella sp. Fabaceae; courtesy of Caian S. Gerolamo. (F) Phloem wedges resulting from atypical cambial activity (single cambium); *Mansoa difficilis*, Bignoniaceae; courtesy of Caian S. Gerolamo. (G) Compound stem originating from atypical procambial patterning, generating a central cylinder and nine peripheral vascular cylinders, each with its own cambium (multiple cambia); *Serjania fuscifolia,* Sapindaceae; courtesy of Robson G. Silva. Images not to scale.

Vascular variants are alternative vascular ontogenies deviating from an ancestral development, achieved through modifications in the organization, activity, position, timing and/or number of vascular meristems. Specifically in stems of seed plants, vascular variants are alternative trajectories that deviate from the typical growth (i.e. eustele + bifacial single cambium; Fig. 1A and B), thereby generating circular or non-cylindrical conformations and altered arrangements of vascular tissues (Fig. 1C–G). These developmental events may originate during primary and/or secondary growth. Therefore, a review of vascular variants originating from procambial origin, and which collectively corroborate a broader developmental based classification of this phenomenon follows.

## Procambium Patterning—and Not Cambial Activity—Generate Vascular Variants in Some Lineages

**Figure 2.**
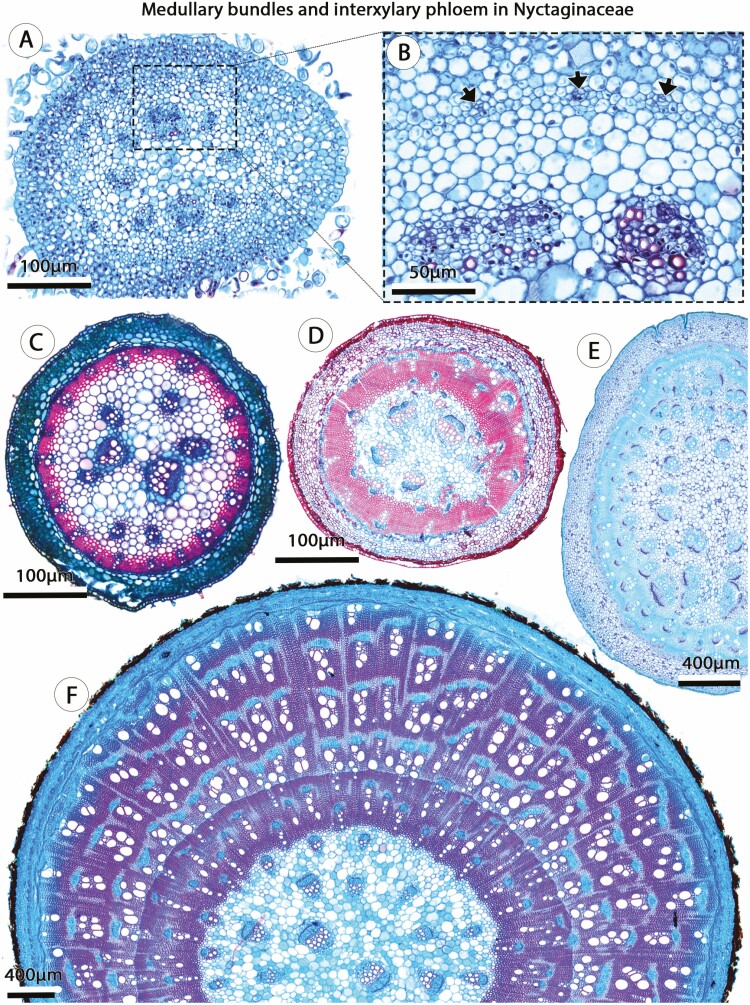
Medullary bundles (procambial variant) and interxylary phloem (cambial variant) in stems of Nyctaginaceae. (A, B). Primary growth with medullary bundles and developing vascular bundles (arrows) derived from the continuous procambium (ring of smaller cells). *Commicarpus scandens.* (C) Early secondary growth showing medullary bundles and cambium developed from the vascular bundles derived from the continuous procambium. *Acleisanthes chenopodioides.* (D) Note that the phloem of vascular bundles derived from the continuous procambium become the first ‘phloem islands’ and, therefore, there is no formation of a continuous vascular cylinder of secondary xylem and secondary phloem. *Guapira pernambucensis.* (E) Medullary bundles and atypical cambial activity producing ‘phloem islands’ within the wood. (F) Adult stem showing medullary bundles with secondary growth, absence of continuous vascular cylinder, and multiple ‘phloem islands’. *Pisoniella glabrata.* (A–F) Light microscopy. (A–D, F) Stained with Safranin and Astra Blue. (E) Stained with Toluidine Blue.

In Nyctaginaceae, three out of four stem ontogenies begin with a modified procambium organization (see Cunha Neto *et al.* 2022). Of these, one ontogeny generates a pattern of ‘interxylary phloem’ (Fig. 2A–F; **see**  **Supporting Information—Table S2**, Glossary) and two ontogenies produce a pattern called ‘successive cambia’ **[see**  **Supporting Information—Fig. S1****]**. In these species, stems are characterized by medullary bundles—which are derived from procambial traces—combined with a continuous procambium that adds a ring of vascular bundles to the primary vascular system called polycyclic eustele (Fig. 2A–C; Cunha Neto *et al.* 2020a). Stems with interxylary phloem show evidence not only of polycyclic eusteles, but also the existence of cambium originating from a continuous procambium. This cambium presents a differential cambial activity since its establishment (Fig. 2C–E) generating phloem strands and associated axial parenchyma within the secondary xylem, collectively called ‘phloem islands’ (Fig. 2D–F; Cunha Neto *et al.* 2021). The phloem of the external vascular bundles of the polycyclic eustele is the first phloem island (Fig. 2C and D), and there is no stage of regular secondary growth (Fig. 2D and F). Therefore, developmental modifications in both procambial and cambial activity are observed in stems with interxylary phloem in Nyctaginaceae. The two other ontogenies that start stem development with polycyclic eusteles develop successive cambia instead of interxylary phloem (**see**  **Supporting Information—Fig. S1**; Cunha Neto *et al.* 2022). Successive cambia in these two cases originate either through an ‘extra-fascicular cambium’ as the first cambium (Cunha Neto *et al.* 2022), or through ectopic cambia—*de novo* cambium formed in an atypical location—after some time of secondary growth has occurred from a cambium in the usual position (Cunha Neto *et al.* 2020b).

**Figure 3.**
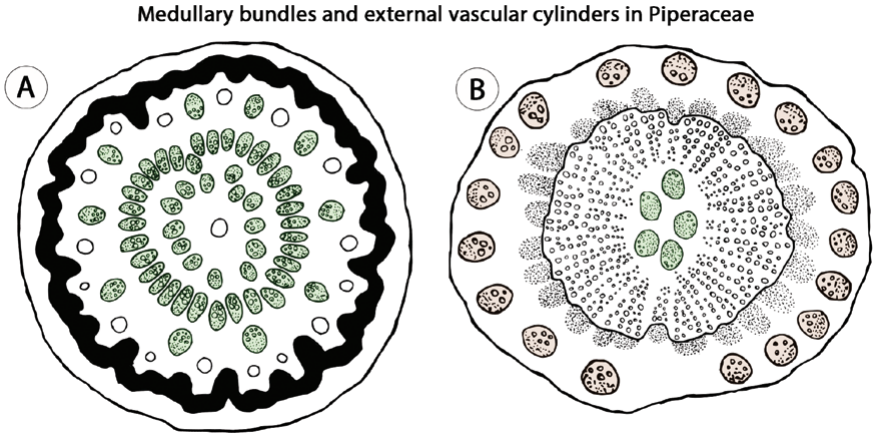
External vascular cylinders in stems of Piperaceae, a case of procambial variant. This ontogeny originates from medullary bundles which undergo secondary growth. (A) Stem during primary growth showing three rings of medullary bundles (three successive coloured rings/green). (B) Mature stem showing medullary bundles in the pith (coloured ring in the center/green), continuous secondary growth derived from a cambium originated from the middle ring of medullary bundles, and isolated ‘external vascular cylinders’ (coloured ring in the periphery/brown) derived from the outer ring of medullary bundles. Small circles indicate vessels in the xylem and dotted area indicates the phloem. Figures not to scale.

In Piperaceae, stem ontogeny of some species initiates with two or more rings of medullary bundles during primary growth (Fig. 3A; Isnard *et al.* 2012). Later in development, some of these species (e.g. *Manekia*) form a bifacial cambium from the middle ring of medullary bundles producing a continuous cylinder of secondary xylem and secondary phloem (Fig. 3B). Each bundle in the external ring experiences secondary growth, whereby each develops into an ‘external vascular cylinder’ (Fig. 3B; Angyalossy *et al.* 2012, 2015). Medullary bundles from the inner ring continue isolated in the pith, which may also undergo secondary growth (Carlquist 2001). This ontogeny indicates that, in Piperaceae, vascular bundles (from primary vasculature) formed in an unusual position are the main precursor of atypical anatomical patterns observed in mature stems.

**Figure 4.**
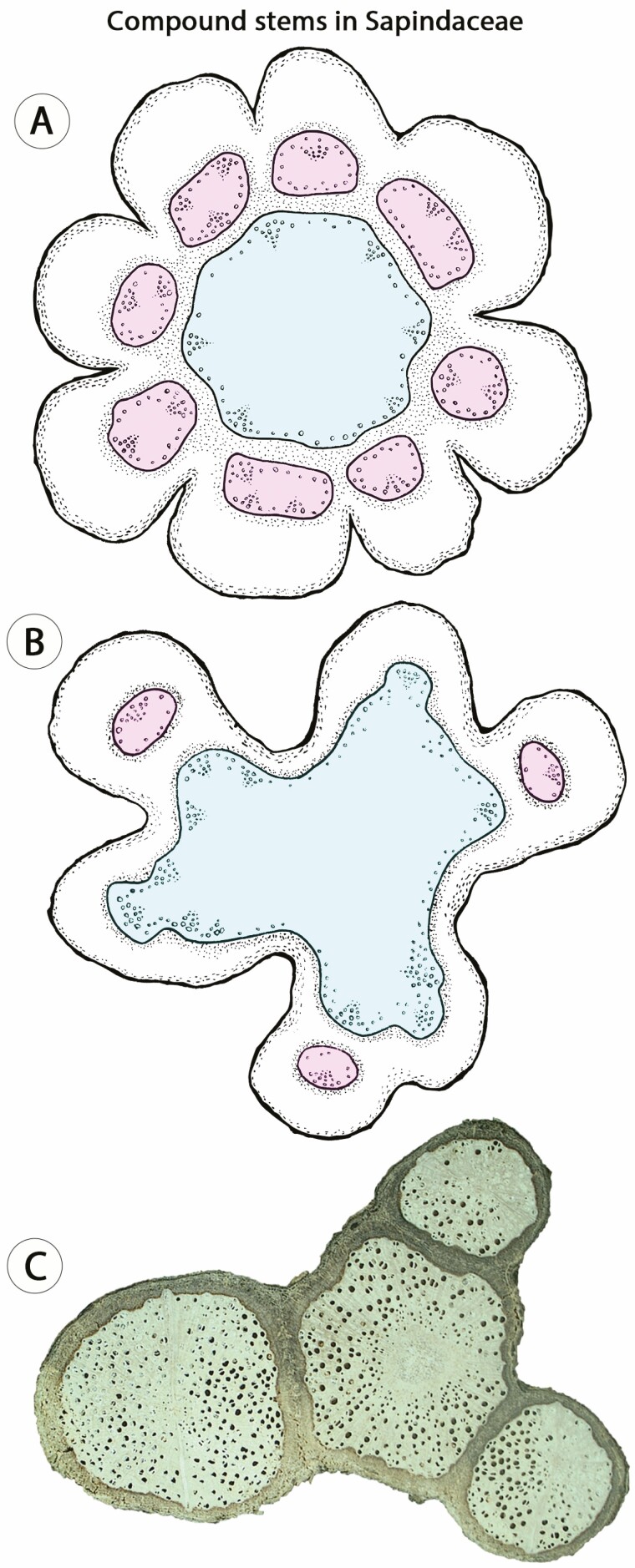
Compound stems in Sapindaceae, a case of procambial variant. (A) Illustration based on stems of many *Serjania* species during transition from primary to secondary growth; each lobe has multiple vascular bundles from which a vascular cambium develops generating a peripheral vascular cylinder (coloured circles in the periphery/pink); a central vascular cylinder is also produced from vascular bundles in the usual position (coloured circle in the center/blue; see also Fig. 1G). (B) Illustration based on stems of many *Paullinia* species during transition from primary to secondary growth; a central vascular cylinder is formed in the usual way (coloured circle in the center/blue), and peripheral vascular cylinders derive from a cambium initiating from a single isolated vascular bundle (coloured circles in the periphery/pink); in this case, three isolated bundles will generate three peripheral vascular cylinders. (C) Mature stem with a central vascular cylinder and three peripheral vascular cylinders. *Paullinia spicata.* Small circles indicate vessels in the xylem and dotted area indicates the phloem. Figures not to scale.

In Sapindaceae, the compound vascular cylinder (= *compound stem*) is the emblematic example of unusual procambial patterning. In general, species with compound stems initiate stem development with lobed stem outlines (Fig. 4A and B). However, two developmental pathways generate similar stems at maturity, both characterized by a central cylinder surrounded by peripheral vascular cylinders (Figs. 1G and 4C). Of these, one ontogeny has two or more vascular bundles organized in a ring-like arrangement (i.e. some bundles have inverted polarity) in each lobe, and from these bundles, a cambium develops and generates the peripheral vascular cylinder (Fig. 4A). Concomitantly, a cambium is formed from vascular bundles in the usual position (delimiting the pith) generating a central vascular cylinder (Fig. 4A). This ontogeny is typical of *Serjania* species, which may produce 3–11 peripheral vascular cylinders (Fig. 1G; Tamaio and Angyalossy 2009). The other ontogeny is typical of *Paullinia* species (Van der Walt *et al.* 1973; Chery *et al.* 2020), whereby peripheral vascular cylinders from a single isolated bundle (instead of various bundles) are formed in the distal portion of the lobes (Fig. 4B). Mature stems of this ontogeny also have a central vascular cylinder produced in the usual way, and generally, only three peripheral vascular cylinders are produced (Fig. 4C).

**Figure 5.**
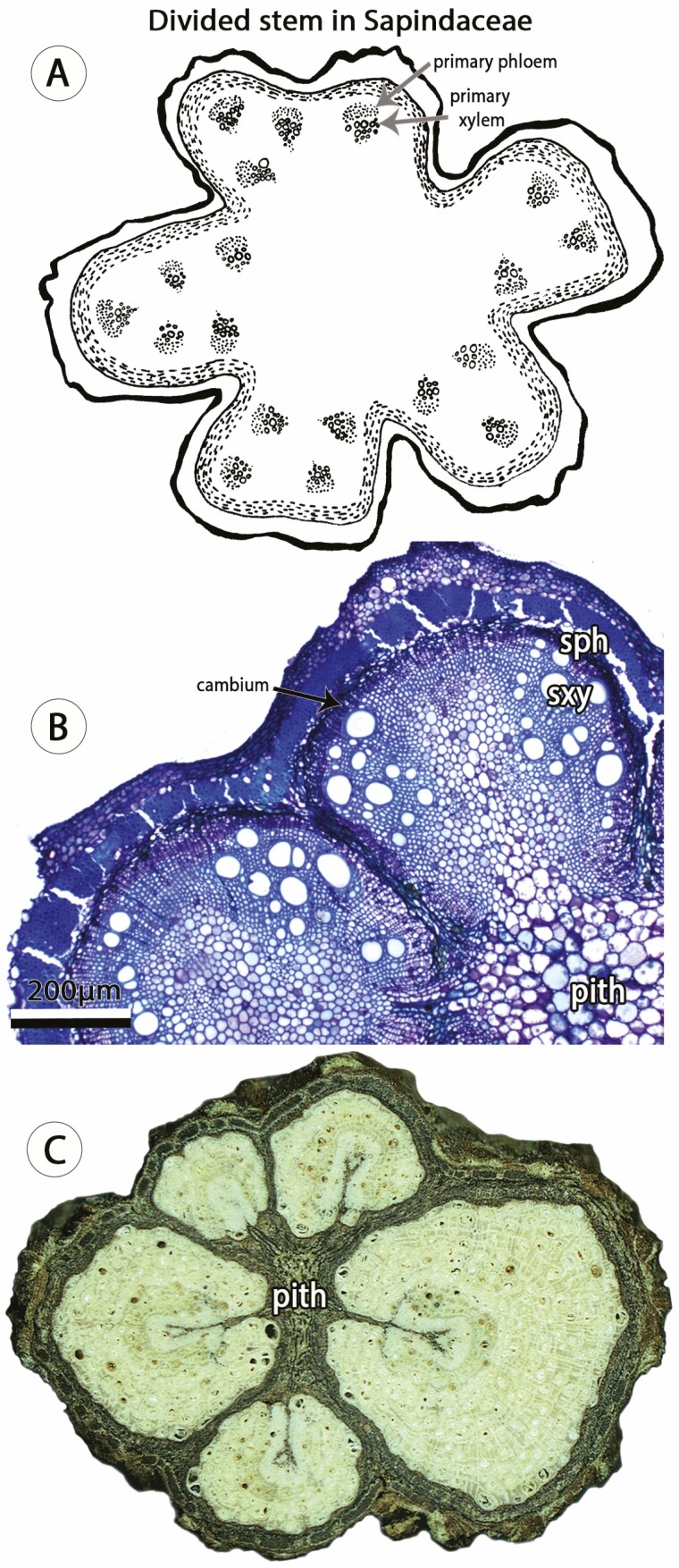
Divided stem in Sapindaceae initiate with atypical procambial patterning. (A) Drawing illustrating young stem with lobed conformation and vascular bundles distributed along the lobes. Figures not to scale. (B) Light microscopy of *Serjania deflexa* showing two cambia generating two peripheral vascular cylinders; each cambium and peripheral vascular cylinder is derived from vascular bundles in one lobe. Stained with Toluidine Blue. Courtesy of Neusa Tamaio. (D) Macroscopic image of adult stem of *Serjania corrugata* showing five peripheral vascular cylinders and no central cylinder. Stem diameter: 15 mm. Courtesy of Robson G. Silva. Small circles indicate vessels in the xylem and dotted area indicates the phloem. sph, secondary phloem; sxy, secondary xylem.

Another example of vascular variants originating from unusual procambial patterning in Sapindaceae is the divided vascular cylinder (= *divided stem*) (Fig. 5A–C). In this type of vascular variant, the eustele is spatially modified through vascular bundles of usual polarity distributed along the lobes and virtually no vascular bundles in the furrows (Fig. 5A). Research suggests that a single cambium may be formed connecting all bundles during the transition from primary to secondary growth (Rizzieri *et al.* 2021), shortly thereafter, the continuous cambial activity is interrupted, generating five circular cambia (one in each lobe; Fig. 5B). In the lobes, each cambium forms a peripheral vascular cylinder (Fig. 5C), and, in some cases, a central cylinder is also formed in late developmental stages (Rizzieri *et al.* 2021).

Taken together, the ontogenies described above (and others discussed below, e.g. ‘intraxylary phloem’) represent developmental modifications that derive from unusual procambial organization, suggesting a distinct origin for alternative vascular ontogenies, which also arise from atypical cambial activity and *de novo* vascular meristems.

## The Three Categories of Vascular Variants

**Figure 6.**
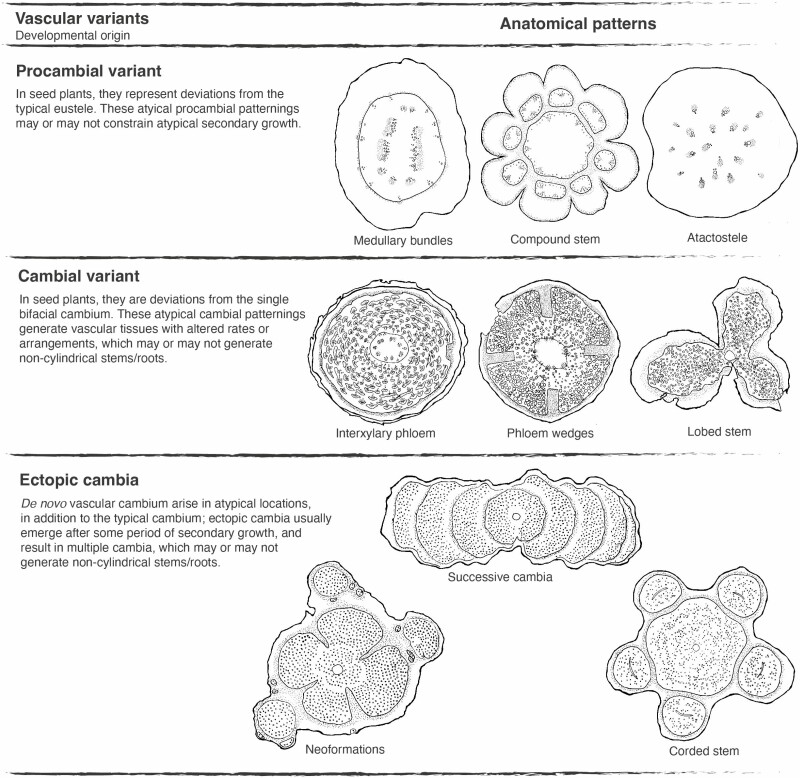
Synthesis of vascular variants categories and their respective anatomical patterns based on the known diversity across seed plants. Only some anatomical patterns are represented in the right side for each category—a complete list of anatomical patterns associated to each category can be found in **Supporting Information—Table S1**. Drawings of anatomical patterns are inspired from cross sections of species of the following families: medullary bundles: Nyctaginaceae, compound stem: Sapindaceae, atactostele: Commelinaceae, interxylary phloem: Nyctaginaceae, phloem wedges: Bignoniaceae, lobed stem: Sapindaceae, neoformations: Rubiaceae, successive cambia: Fabaceae, corded stem: Sapindaceae. Small circles indicate vessels in the xylem and dotted area indicates the phloem.

Following this developmental approach, three categories of vascular variants are considered: (i) *procambial variants* (inspired by Lopes *et al.* 2017), (ii) *cambial variants* (following Carlquist 2001) and (iii) *ectopic cambia* (following Spicer and Groover 2010). Each of these three developmental based categories generates numerous anatomical patterns (Fig. 6; **see**  **Supporting Information—Table S3**).

Since cambial activity is not the only the source of atypical vascular ontogenies, the term ‘cambial variants’ fails to accurately describe the breadth of alternative vascular development in plants. This terminological inadequacy has been noted by other researchers for some time, including Dr. Sherwin Carlquist, who expressed the inadequacy of the term ‘cambial variants’ when discussing some Sapindaceae (pers. comm. to Dr. Neusa Tamaio). When describing *compound stems* of the Sapindacee family, three terms have previously been utilized in place of ‘cambial variants’: (i) ‘polystelic stem’ (Acevedo-Rodríguez 1993), (ii) ‘multistelar stem’ (Klaassen 1999) and (iii) ‘procambial variant’ (Lopes *et al.* 2017). The first two terms were disputed by Tamaio and Angyalossy (2009) on the basis that a single (and not multiple) stele ise produced, whilst the term ‘procambial variant’, arguably a more adequate term to describe compound stems, has not flourished and has only been repeated in related literature once (Pace *et al.* 2022). Interestingly, besides compound stems in Sapindaceae (Lopes *et al*. 2017), previous studies have also considered medullary bundles a type of ‘cambial variants’ (de Bary 1884; Esau 1965; Costea and DeMason 2001; Beck 2010), emphasizing the contribution of procambial modifications to the interpretation of alternative vascular ontogenies in seed plants.

**Figure 7.**
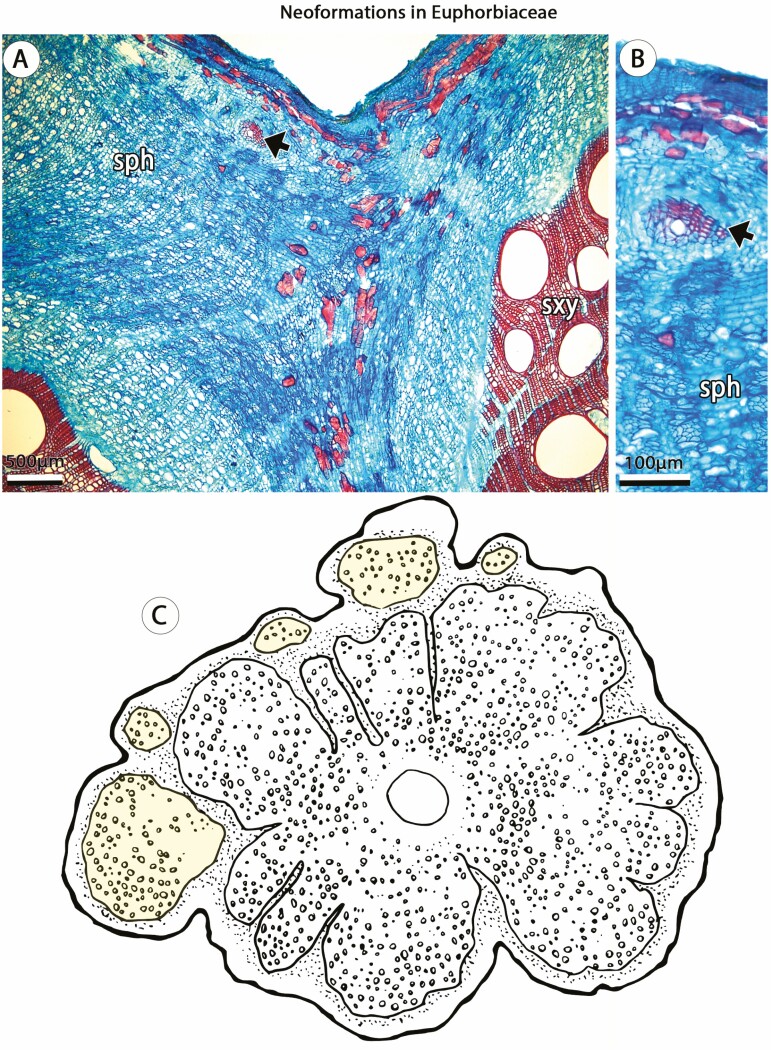
Neoformations in stems of *Dalechampia* (Euphorbiaceae), a case of ectopic cambia. (A, B) *Dalechampia alata.* (A) Adult stem showing the initiation of an ectopic cambia (arrow) from vascular parenchyma at the periphery of the non-conducting phloem. (B) Detail of ectopic cambia (arrow) initiation. (C) Line drawing of mature stem showing multiple ectopic cambia generating neoformations (yellow) at the periphery of the vascular cylinder. Small circles indicate vessels in the xylem and dotted area indicates the phloem. sph, secondary phloem; sxy, secondary xylem. (B, C) Stained with Safranin and Astra Blue.

Understanding the origin of complex ontogenies is critical since similar mature stems may develop through distinct ontogenetic processes (Cabanillas 2016). For instance, in Sapindaceae, three major stem patterns are known to produce a ‘cable-like’ structure (e.g. compound, divided and corded), revealing the possession of multiple vascular cylinders (Tamaio *et al.* 2011). Although generating similar adult forms, these anatomical patterns derive from both procambial variants (e.g. compound stems, Fig. 4) and ectopic cambia (e.g. corded stem, Fig. 6). Another example involves ‘external vascular cylinders’ described for distantly related angiosperms families, including Bignoniaceae, Combretaceae, Euphorbiaceae, Piperaceae, Rubiaceae and Sapindaceae (Angyalossy *et al*., 2012, 2015) and in which the ‘external vascular cylinders’ derived from the activity of both primary and secondary meristems. In Piperaceae, this pattern originates as a procambial variant, in which isolated medullary bundles undergo secondary growth generating the ‘external vascular cylinders’ (Fig. 3). This developmental pathway, although somewhat similar, is not comparable to the development of most anatomical patterns of Sapindaceae or the phenomenon observed in Rubiaceae (Fig. 7; Leal *et al.* 2020) or Euphorbiaceae (Fig. 7), which are derived from ectopic cambia. These observations indicate that developmental based categories are fundamental for a better understanding of vascular diversity in seed plants.

Undoubtedly, vascular variants are complex anatomies, and like the boundaries between procambium and cambium (Esau 1965), their ontogenetic stages cannot be sharply distinguished in some cases. This fuzziness has culminated in contrasting interpretations. For instance, the distinctive stem anatomy of *Aristolochia* (Piperales)—with wide, large rays greatly dividing the axial elements of the vascular system into discrete portions—was considered a typical growth by Esau (1965) but is a pattern of ‘cambial variants’ in several recent studies (i.e. axial vascular elements in segments, *sensu*  Angyalossy *et al*. 2015). Other patterns with atypical ray development, as well as raylessness (reviewed by Frankiewicz and Oskolski 2023), may represent additional examples. Moreover, vascular variants can be constrained by the activity of other primary meristems. This is observed, for instance, in stems that develop as non-cylindrical organs since early stages, as observed in Malvaceae (Luna-Márquez *et al*. 2021) or Sapindaceae (Chery *et al*. 2020). However, non-cylindrical stems are frequently determined by cambial variants (e.g. lobed stem) or ectopic cambia (e.g. successive cambia), generating different categories (e.g. angular, flattened, lobed; Luna-Márquez *et al*. 2021).

## Revisiting Alternative Ontogenies in Seed Plants

An investigation of vascular ontogenies following the present developmental framework reveals distinct categories of vascular variants in both stems and roots of major lineages of seed plants. For instance, there is a striking stele variation in stems of some Nymphaeales (ANA grade) and Gunnerales (core eudicots), including polycyclic eusteles and siphonostele (Seago 2020; Seago *et al.* 2021). Mature stems of Apocynaceae (asterids, eudicot) have been reported with ‘*intra*xylary phloem’, *inter*xylary phloem, successive cambia and non-cylindrical stems (Acevedo-Rodríguez 2015, onwards; Angyalossy *et al.* 2015), while young stems are described with siphonosteles, bicollateral bundles and/or medullary bundles (Salas *et al.* 2018; Sathya *et al.* 2022). This indicates that not only cambial variants occur in this family, but also procambial variants and ectopic cambia, which may or may not be developmentally constrained.

The typical stem development of monocots could also be described as vascular variants since they deviate from the putative ontogeny in the ancestor of seed plants (Fig. 6). Monocots are characterized by the well-known ‘atactostele’ (Fig. 1C), which is considered as a highly modified eustele (Beck *et al.* 1982), closed vascular bundles (absence of procambial remnants) and by the lack of a ‘typical cambium’. However, some lineages produce secondary growth through the Secondary Thickening Meristem (STM), also called monocot cambium (Carlquist 2012). In these lineages (e.g. some Asparagales: Jura-Morawiec *et al.* 2015; and Arecales, also through the STM and not diffuse growth: Botânico and Angyalossy 2013), the monocot cambium produces vascular bundles centripetally and parenchyma centrifugally. Although considered analogous meristems, the monocot cambium and the typical cambium of seed plants show a considerable overlap of gene expression (Zinkgraf *et al.* 2017; Roodt *et al.* 2019; Povilus *et al.* 2020). These observations indicate that similarities in molecular programmes between the two lineages were likely achieved through the co-optation of key genes during the evolution of the monocot cambium (Zinkgraf *et al.* 2017). It also highlights the advantage of broad developmental based categories allowing the inclusion of monocots in the context of alternative vascular ontogenies, which may facilitate integrative research exploring vascular diversity in flowering plants.

In roots, vascular variants are understood to be the cause for the concentric rings in sugar beets (Chenopodiaceae) due to successive cambia—ectopic cambia (Artschwager 1926). Vascular variants are also observed in roots of species from other families of gymnosperms (e.g. successive cambia in *Cycas revoluta*: Avetta 1887) and angiosperms (Schenck 1893), including Nyctaginaceae (Cunha Neto *et al.* 2020b) and Polygonaceae (Rajput 2015), also with successive cambia, Bignoniaceae with phloem wedges (Victorio 2016) or Apiaceae forming strands of vascular tissues called ‘multisteles’ (Chuang 1970). Procambial variants in roots of seed plant lineages appear to be less common than those found in their respective stems, though the variants may be present in parasitic plants (Kuijt and Bruns 1987; Carlquist 1988; Wilson and Calvin 1996; Pellissari *et al*. 2022). As emphasized by Wilson and Calvin (1996: 104), in parasitic plants, alternative ontogenies ‘are problematic as they may refer to anomalous primary growth’ instead of cambial modifications. The vascular variant concept would also be appropriate to describe these alternative ontogenies.

## Phylogenetic Distribution of Vascular Variants

**Figure 8.**
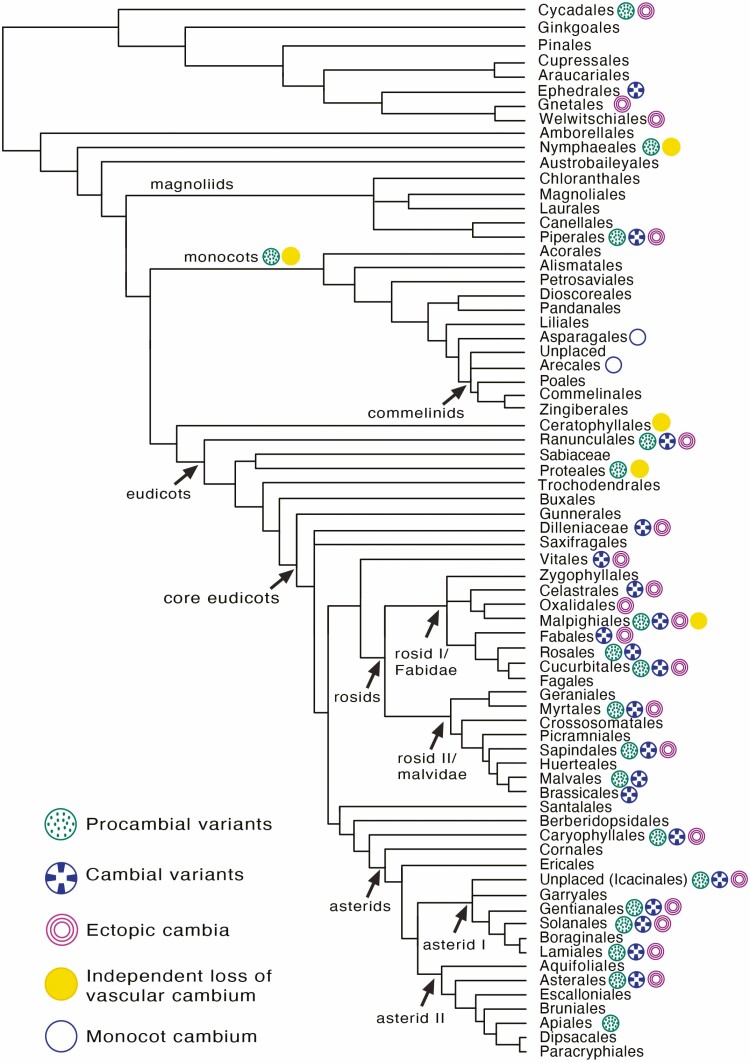
Distribution of vascular variants in the phylogeny of seed plants. Tree topology follows Stevens (2001 onwards) for angiosperms and Li *et al.* (2021) for gymnosperms. All types of ‘cambial variants’ presented by Angyalossy *et al.* (2015) are distributed in one of the three categories of vascular variants **[see**  **Supporting Information—Table S2** for additional details**]**. The ‘monocot cambium’ is indicated for their respective orders and the atactostele is indicated in the branch leading to the monocot lineage.

Vascular variants have evolved independently multiple times across the evolution of seed plants. *Procambial variants*, *cambial variants* and *ectopic cambia* are reported in at least 24, 21 and 22 orders, respectively (Fig. 8). In total, vascular variants occur in 4 orders of gymnosperms and 31 orders of angiosperms (Fig. 8; **see**  **Supporting Information—Table S4**). Vascular variants are disproportionately distributed, occurring more abundantly in angiosperms in comparison to gymnosperms, and more frequently in eudicots when compared to other flowering plants (Fig. 8). Outside the core eudicots, Piperales are distinct for having all three categories represented in the order (Fig. 8). Monocots produce both procambial variants and species with the monocot cambium. Within gymnosperms, vascular variants are characterized predominantly by successive cambia (ectopic cambia). Nevertheless, procambial variants (i.e. medullary bundles) and cambial variants (e.g. non-cylindrical) are also observed in Cycadales and Ephedrales, respectively **[see**  **Supporting Information—Table S4****]**.

## Developmental Implications of the Vascular Variants’ Framework

Within seed plants, over 10 anatomical patterns of vascular variants have been described as types of ‘cambial variants’ (Bodnar and Coturel 2012; Yang *et al.* 2014; Acevedo-Rodríguez 2015 onwards; Angyalossy *et al.* 2015). Here, these anatomical patterns are further grouped into one of three vascular variants categories, which emphasizes their developmental origin (Fig. 6; **see**  **Supporting Information—Table S3**). Although naming conventions previously employed to describe anatomical patterns vary and are not developmentally accurate, they nonetheless should remain in certain instances, as they carry important taxonomic meaning (e.g. Sapindaceae).

In a sense, procambial variants and ectopic cambia encompass changes in the location and spatial organization of vascular meristems (and their products), while cambial variants are comprised of changes primarily in the rate and spatial distribution of cambial derivatives (Fig. 6). Multiple anatomical patterns may also be described in a single stem, which result from a combination of two or more vascular variants categories. In Nyctaginaceae, for instance, stems with procambial variants (i.e. medullary bundles) independently produce distinct vascular variants during secondary growth, for example, interxylary phloem (cambial variants) or successive cambia (ectopic cambia). In other cases, such as the ‘external vascular cylinders’ in Piperaceae (Fig. 3) and the compound stems in Sapindaceae, the modifications during primary growth (procambial variants) developmentally constrain the atypical anatomical pattern observed during secondary growth (Fig. 4).

Acceptance of the term vascular variants as an overarching developmental based framework for interpreting alternative vascular ontogenies affords the opportunity to review fundamental phenomena in plant biology, such as ectopic cambia. Ectopic cambia, which comprise the formation of a *de novo* cambium in addition to the typical cambium, arise from parenchymatous tissues derived from the ground meristem (i.e. the cortex—Leal *et al*. 2020; Nejapa *et al.* 2021) or from vascular tissue, including the pericycle (Tamaio *et al*. 2009; Cunha Neto *et al.* 2018), phloem axial parenchyma (Leme *et al.* 2020), phloem rays (Cunha Neto *et al.* 2018), non-lignified axial parenchyma in the xylem (Pace *et al.* 2018) and pith cells (Carlquist 2001; Patil and Rajput 2008). At the anatomical level, ectopic cambia include patterns delimiting concentric rings or discrete fragments, that is, successive cambia (Tamaio *et al*. 2009;  Cunha Neto *et al.* 2018), and circular vascular units, that is, neoformations or neo-formed vascular strands (Bastos *et al.* 2016; Leal *et al*. 2020), which includes the typical peripheral cylinders in corded stems of *Thinouia*, Sapindaceae (Tamaio and Somner 2010). Independent of their anatomical organization, these patterns derive from the same developmental mechanism, thus, constituting examples of the same vascular variant category (Fig. 6). In the context of vascular variants, ‘neoformations’ are used particularly to describe additional vascular tissue produced in late developmental stages of woody vines containing other vascular variants (e.g. Bignoniaceae: Angyalossy *et al.* 2015; Malpighiaceae: Cabanillas *et al.* 2017; Sapindaceae: Bastos *et al.* 2016). Because ‘neoformations’ normally occur in late developmental stages, this phenomenon was initially characterized as ‘tertiary growth’ (Van Tieghem 1884), also called ‘tertiary thickening’—used to characterize anatomical modifications in storage organs such as in potato or beets (Hayward 1938; Wilson and Lowe 1973; Forbes 1985). ‘Tertiary growth’ is, therefore, ambiguous as it includes vascular (e.g. ectopic cambia in beets) and non-vascular processes (e.g. parenchyma proliferation in potato; Tribble *et al.* 2021).

Another phenomenon worth considering is conducting vascular tissue in the pith. There are distinct patterns that originate from developmentally diverse processes and which are described with several names, for example, ‘internal phloem’ (Patil and Rajput 2008; Rajput and Gondaliya 2017), bicollateral bundles (Hayden and Hayden 1994; Patil *et al.* 2011), perimedullary phloem (Araújo and Costa 2006) and medullary cambia (Philipson and Ward 1965). In the context of vascular variants, these overlapping yet disparate phenomena (Carlquist 2012) have been placed under the term ‘*intra*xylary phloem’ (Carlquist 2013a; Angyalossy *et al.* 2015; Rajput *et al.* 2022), and considered a synapomorphy of some lineages such as Myrtales and core Convolvulaceae (Stevens 2001 onwards; Stefanovic *et al.* 2002). Since many patterns deviate from the typical eustele, for instance, through the formation of bicollateral bundles (e.g. Cucurbitaceae) or siphonosteles (e.g. Apocynaceae), they are better described as cases of procambial variants. In some cases, perimedullary pith cells may differentiate into phloem followed by a *de novo* cambium, which may produce large amounts of vascular tissue in the pith (Rajput *et al.* 2018, 2022). Yet, in some cases, it may be difficult to distinguish whether perimedullary phloem originates from procambial-derived cells or from a *de novo* cambium, in which cases even vascular variants categories may be difficult to apply. Fossil plants may also pose a difficult scenario for determining if conducting vascular tissue in the pith originated from procambial remnants or ectopic cambia. This difficulty may further explain the use of the term ‘medullary vascular system’ (Bodnar and Coturel 2012).

Remarkably, ectopic cambia generate enormous vascular diversity representing a striking developmental potential that may be present in many but not all plant lineages. While their structural diversity and functional significance have begun to be elucidated (Schmitz *et al.* 2008; Carlquist 2013b; Robert *et al.* 2014), and possible mechanisms for the origin have been proposed (Robischon *et al*. 2011; Smetana *et al*. 2019), the molecular underpinnings of this phenomenon await thorough clarification.

## Vascular Variants Beyond Seed Plants

It is believed that the fossil record likely encompasses a much broader diversity of vascular ontogenies if compared to extant flora (Decombeix *et al*. 2019). Since alternative vascular ontogenies also exist in other extant and fossil tracheophytes, it may be possible to apply a broader context to the vascular variants’ framework. For example, in free-sporing plants, two instances of deviations from typical to atypical ontogenies in relation to their putative ancestors can be found in the independent evolution of the unusual bifacial cambium in *Isoëtes* (Lycophyta; Spicer and Groover 2010; Onyenedum and Pace 2021), and the unifacial cambium in some Ophioglossales (ferns; Esau 1965; Stevenson 1980). Of note, the instance of unifacial cambium in Ophioglossales is debated (Rothwell and Karrfalt 2008). Undeniably, most cases of vascular variants in stems of fossil and extant ferns should be related to procambial modifications as they display an enormous diversity of stele types including protosteles and siphonosteles, which allowed for their rapid radiation during the Carboniferous period (Suissa and Friedman 2022). Debate in this field has promoted significant advances in stelar theory (Tomescu 2021).

Deviations from typical to atypical ontogenies are also prolific in the fossil record (Artabe and Brea 2003; Bodnar and Coturel 2012; Crepet and Niklas 2019; D'Antonio and Boyce 2021). In fossil seed plants, all three categories of vascular variants can be recognized. ‘Medullary vascular systems’ (Corystopermales: Bodnar 2012) likely include cases of procambial variants, as in the atypical medullosan eustele (Dunn *et al.* 2003). Stems with differential cambial activity that generate ‘axial vascular elements in segments’ (Corystopermales: Bodnar and Coturel 2012) represent one type of cambial variant, while successive cambia (e.g. fossil Cycadales: Artabe and Stevenson 1999; Bodnar and Coturel 2012) and ‘neoformations’ (e.g. Sapindaceae: Jud *et al*. 2021) indicate the existence of ectopic cambia. This evidence suggests an enduring use of vascular variants, and highlights their systematics, functional and ecological significance in the evolution of plants. A greater understanding of the diversity beyond that of seed plants and fossil plants is needed, but beyond the scope of this study. Additional research is necessary, with respect to fossil and non-seed plant lineages, in order to expand the framework towards a more robust inclusion of all vascular plants.

## Evolutionary Implications of Vascular Variants

Like other complex traits, the convergent evolution of vascular variants across seed plants is a striking phenomenon worth considering. In the past few years, research on this topic has focused on understanding not only vascular variants’ developmental anatomy, but also their evolutionary history (e.g. Bignoniaceae: Pace *et al.* 2009; Malpighiaceae: Quintanar-Castillo and Pace 2022; Malvaceae: Luna-Márquez *et al.* 2021; Sapindaceae: Chery *et al.* 2020), their impact to lineages diversification (e.g. Nyctaginaceae: Cunha Neto *et al.* 2022), structure–function relationships (Feild and Isnard 2013; Rowe and Speck 2015; Gerolamo *et al.* 2020; Hu and Lin 2022) and molecular regulation (Lima 2020), which remains largely unknown.

Since vascular variants are abundantly distributed in lineages containing climbing plants (Angyalossy *et al*. 2015), their ecological and evolutionary significances have been tested more frequently. Studies suggest that vascular variants evolved in association with the evolution of the climbing habit in several families (e.g. Bignoniaceae: Pace *et al.* 2009; Malpighiaceae: Quintanar-Castillo and Pace 2022). Along with their disparate climbing mechanisms (Isnard and Silk 2009; Gianoli 2015), the evolution of vascular variants in climbing plants likely facilitated their ecological diversification and physiological performance. Such complex anatomies are believed to increase stem flexibility, conductivity and mechanical resistance allowing them to climb without breaking apart (Feild and Isnard 2013; Isnard and Feild 2015). Furthermore, the evolution of vascular variants has been correlated with lobed conformation in primary growth (e.g. Sapindaceae: Chery *et al.* 2020), the arrangement of the primary vascular system and the formation of dermal appendices, such as prickles (e.g. Malvaceae: Luna-Márquez *et al.* 2021). Complex anatomies are further observed in fossil climbing plants dating from the Carboniferous period, such as the seed fern *Medullosa* (Dunn *et al.* 2003), and they are also recorded in early Miocene *Ampelorhiza*, the oldest fossil evidence of Paullinieae, Sapindaceae (Jud *et al.* 2021).

By contrast, vascular variants are widespread in some lineages regardless of growth forms (e.g. Menispermaceae: Jacques and de Franceschi 2007; Nyctaginaceae: Cunha Neto *et al.* 2022). In Nyctaginaceae, which have diverse growth forms and whose most common ancestor is reconstructed with vascular variants, increased diversification rates have been associated with the acquisition of medullary bundles, but not with transitions from self-supporting to climbing plants (Cunha Neto *et al.* 2022). The evolution of the climbing habit has been postulated as a key innovation in angiosperms (Gianoli 2004). Nyctaginaceae is likely one of a few examples where Gianoli’s (2004) hypothesis did not sustain. Among other evidence, support for Gianoli’s hypothesis can be found in Piperales, which is also diverse in growth forms and cambial modifications (Trueba *et al.* 2015), including vascular variants (Angyalossy *et al*. 2015). The ancestor of the perianth-bearing Piperales was reconstructed with a herb- or shrub-like habit, and the climbing habit was proposed as a derived growth form, which might have been a key feature in the diversification of *Aristolochia*, the most speciose lineage and the only genus with climbing plants (Wagner *et al.* 2014).

In self-supporting plants (non-climbing), functional properties of vascular variants may include adaptations for storage (e.g. beets: Artschwager 1926) or strategies for enhancing survival in conditions of extreme physiological drought such as arid soils or in the mangrove environment (e.g. *Avicennia*, the mangrove tree: Robert *et al.* 2014). Alternatively, vascular variants have been interpreted as evolutionary constraints (phylogenetic inertia), which means that self-supporting plants inherited these complex morphologies from their climbing ancestors in which these features could have been originally selected for the climbing habit (Jacques and de Franceschi 2007; Pace 2015; Gerolamo and Angyalossy 2017). Characterizing the vasculature of predominantly herbaceous plants, the atactostele in monocots is a remarkable deviation with enormous implications that explain some of the growth habit differences between monocots and eudicots (DeMason 1994). Remarkably, radial growth through the monocot cambium evolved solely in two lineages where arborescent monocotyledons are conspicuous. However, similar radial growth has also been reported in rhizomes and corms of some geophytes, especially in the Asparagales (Tribble e*t al*. 2021).

In evolutionary developmental biology, any genetic change resulting in altered phenotypes may be considered a shift in developmental programmes which can modify the descendant’s morphology relative to the putative ancestor (Bateman 1994). In vascular development, evidence suggests that distinct developmental genetic modules can be protracted, prolonged, change location or acquire new functions resulting in modifications in cambial development (Onyenedum and Pace 2021). These re-patterning mechanisms likely occur through modifications in expression patterns of homeotic genes that determine shared molecular regulatory programmes across vascular plant lineages (Tomescu and Groover 2019). Enhancing our understanding of gene function in stem development of plants with and without vascular variants will provide insights into lineage-specific regulators, as well as conserved molecular programmes in vascular meristem formation (Tomescu and Groover 2019). In other words, the convergent evolution of vascular variants may be due to modifications in the tempo and mode of gene regulatory programmes generating the multitude of anatomical patterns observed in plants. Therefore, within a developmental approach, the identification of developmental origins and processes determining vascular variants may be informative at various biological scales with meaningful evolutionary implications.

## Concluding Remarks

Anatomical patterns resulting from modifications to the origin, development and activity of vascular meristems in comparison to a putative ancestor of a given lineage are here called ‘vascular variants’. This term reflects that vascular ontogenies can be altered through re-patterning of both primary and secondary vascular meristems, as well as through the natural formation of ectopic cambia. In this context, ‘vascular variants’ unequivocally describe the diversity of alternative vascular ontogenies in seed plants, as opposed to historical yet vague terms such as ‘anomalous secondary growth’ and ‘cambial variants’. Rooted in an evolutionary and developmental context, this developmental based framework reveals three categories of vascular variants: *procambial variants*, *cambial variants* and *ectopic cambia*. Each category is defined by the origin of its vascular meristem and comprises multiple anatomical patterns. Each named anatomical pattern may be further formed through relatively distinct ontogenies, yet they are predictably conserved within a given species. Anatomical patterns have important systematic value and, therefore, precisely clarifying the diverse origins of such complex anatomies will facilitate our communication about the evolution of vascular meristems and potentially reduce barriers to research. As we strengthen our understanding of the developmental origins and ontogenetic processes generating vascular variants, future studies are warranted, and may prove essential to our understanding of molecular regulation in these complex anatomies. These works will be necessary not only to shed light on the diversity of vascular variants at the molecular level but will also elucidate the underlying mechanisms shaping the existing diversity of vascular structures in plants.

## Supporting Information

The following supporting information is available in the online version of this article –

Table S1. List of terms used to describe vascular variants in different languages.

Table S2. Glossary of terms related to vascular variants.

Table S3. List of anatomical patterns distributed across categories of vascular variants as observed in seed plants.

Table S4. Distribution of vascular variants and anatomical patterns across orders and families of seed plants.

Figure S1. Ontogenies generating procambial variants and successive cambia in Nyctaginaceae.

plad036_suppl_Supplementary_Figure_S1

plad036_suppl_Supplementary_Tables

## Sources of Funding

No direct funding is associated with this manuscript. Previously, the author received financial support from Coordenação de Aperfeiçoamento de Pessoal de Nível Superior – Brasil (CAPES) – Código de Financiamento 001 and Fundação de Amparo à Pesquisa do Estado de São Paulo (FAPESP) (2017/17107-3). Current research is supported by Startup lab funds from Cornell University.

## Contributions by the Authors

I.L.C.N designed and wrote the manuscript.

## Data Availability

The original contributions presented in the study are included in the Supporting Information and in online repositories [Zenodo: 10.5281/zenodo.8003032 and GitHub: github.com/ilcneto/VascularVariants]. Further inquiries can be directed to the corresponding author.

## Notes

**Form and Function. Chief Editor:** Kate McCulloh
