# Correction to: Vascular variants in seed plants—a developmental perspective

**DOI:** 10.1093/aobpla/plad050

**Published:** 2023-08-05

This is a correction to: Israel L Cunha Neto, Vascular variants in seed plants—a developmental perspective, AoB PLANTS, Volume 15, Issue 4, July 2023, plad036, https://doi.org/10.1093/aobpla/plad036

In this manuscript, the legend of Figure 1E has been corrected.

Upon original publication, Figure 1E legend was given as follows: ‘(E) Asymmetrical stem resulting from atypical cambial activity (single cambium); *Schnella* sp. Fabaceae; courtesy of Caian S. Gerolamo.’

The legend has been corrected to read:

(E) Non-cylindrical stem resulting from atypical cambial activity (single cambium); *Schnella* sp. Fabaceae; courtesy of Caian S. Gerolamo.’

