# Supplementary figures and images for: Vascular variants in seed plants—a developmental perspective
Source: AoB Plants. 2023 Jul 12;15(4):plad036. doi: 10.1093/aobpla/plad036 (PMC10355320; doi:10.1093/aobpla/plad036)

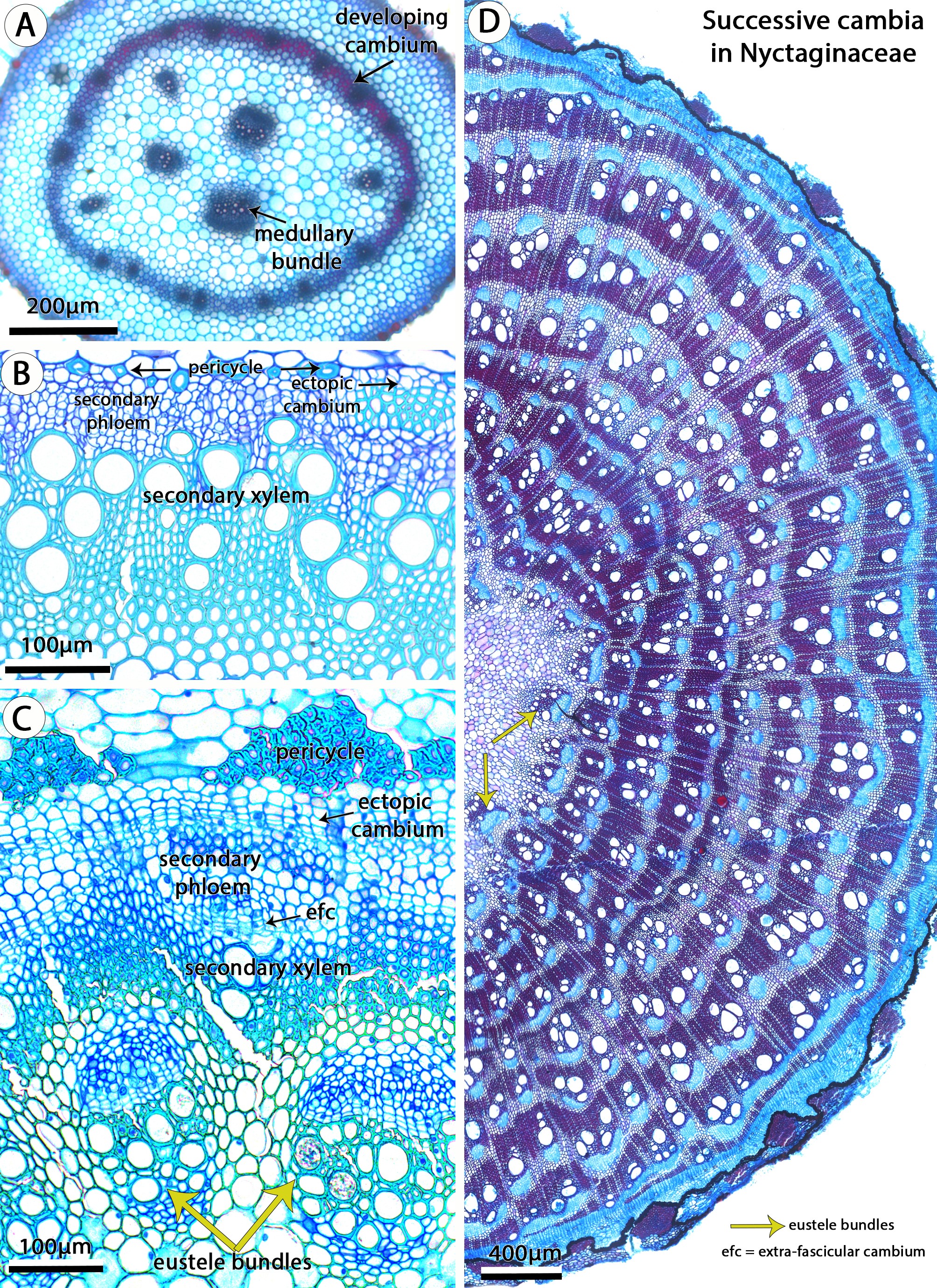

Supplement: plad036_suppl_Supplementary_Figure_S1 [file plad036_suppl_supplementary_figure_s1.jpeg]
